# Feather-like Gold Nanostructures Anchored onto 3D Mesoporous Laser-Scribed Graphene: A Highly Sensitive Platform for Enzymeless Glucose Electrochemical Detection in Neutral Media

**DOI:** 10.3390/bios13070678

**Published:** 2023-06-25

**Authors:** Achraf Berni, Aziz Amine, Juan José García-Guzmán, Laura Cubillana-Aguilera, José María Palacios-Santander

**Affiliations:** 1 Laboratory of Process Engineering and Environment, Faculty of Sciences and Techniques, Hassan II University of Casablanca, P.A. 149, Mohammedia 28810, Morocco; achrafberni@gmail.com; 2 Department of Analytical Chemistry, Institute of Research on Electron Microscopy and Materials (IMEYMAT), Faculty of Sciences, Campus de Excelencia Internacional del Mar (CEIMAR), University of Cadiz, Campus Universitario de Puerto Real, Polígono del Río San Pedro S/N, 11510 Puerto Real, Cádiz, Spain; juanjogg91@gmail.com (J.J.G.-G.); laura.cubillana@uca.es (L.C.-A.); josem.palacios@uca.es (J.M.P.-S.)

**Keywords:** non-enzymatic detection, laser-scribed graphene, gold nanostructures, neutral medium, disposable sensor, glucose sensor

## Abstract

The authors present a novel sensing platform for a disposable electrochemical, non-enzymatic glucose sensor strip at physiological pH. The sensing material is based on dendritic gold nanostructures (AuNs) resembling feather branches, which are electrodeposited onto a laser-scribed 3D graphene electrode (LSGE). The LSGEs were fabricated via a one-step laser scribing process on a commercially available polyimide sheet. This study investigates several parameters that influence the morphology of the deposited Au nanostructures and the catalytic activity toward glucose electro-oxidation. The electrocatalytic activity of the AuNs-LSGE was evaluated using cyclic voltammetry (CV), linear sweep voltammetry (LSV), and amperometry and was compared to commercially available carbon electrodes prepared under the same electrodeposition conditions. The sensor demonstrated good stability and high selectivity of the amperometric response in the presence of interfering agents, such as ascorbic acid, when a Nafion membrane was applied over the electrode surface. The proposed sensing strategy offers a wide linear detection range, from 0.5 to 20 mM, which covers normal and elevated levels of glucose in the blood, with a detection limit of 0.21 mM. The AuNs-LSGE platform exhibits great potential for use as a disposable glucose sensor strip for point-of-care applications, including self-monitoring and food management. Its non-enzymatic features reduce dependence on enzymes, making it suitable for practical and cost-effective biosensing solutions.

## 1. Introduction

Glucose, a vital biological molecule, plays a crucial role in the metabolism of all living organisms [1]. Its precise and sensitive monitoring is essential, including the food and beverage industry [2] and clinical diagnostics [3]. Glucose sensing in urine and blood samples is commonly employed in the screening and management of diabetes [4]. The typical range for glucose concentration is from 0 to 0.8 mM in urine and 3 to 8 mM in blood [5]. By the year 2030, it is estimated that the worldwide diabetic population will exceed 400 million individuals [6]. Consequently, in the absence of a definitive cure for diabetes, glucose monitoring is undeniably critical for effective diabetes management. Extensive efforts have been dedicated to the development and commercialization of glucose sensors for convenient self-monitoring at home.

While various analytical techniques are available for detecting glucose, such as colorimetric methods [7], electrochemical sensors remain one of the most effective tools due to their portability, high sensitivity, low cost, and simplicity of instrumentation [8]. Currently available glucose sensors in the market predominantly rely on the use of enzymes, particularly glucose oxidase (GOx). These enzymes are typically immobilized on the electrode surface to catalyze the glucose reaction, enhancing the sensitivity and specificity of the biosensor [9,10]. However, they can be expensive and susceptible to degradation or denaturation, limiting their performance and lifespan in challenging conditions such as high temperature, humidity, and extreme pH values [11,12]. To address these limitations, non-enzymatic glucose detection emerged as a growing area of research, offering the potential for highly sensitive and selective glucose sensors utilizing nanomaterials [13,14,15,16].

The glucose sensors currently available in the market primarily rely on electrical sensing signals, enabling direct readout without the need for additional signal processing. This feature offers benefits for downsizing sensing components [17,18]. In this context, screen-printed electrodes (SPEs) are frequently employed as electrode strips due to their affordability and ability to be manufactured in large quantities. Nevertheless, SPEs do have certain drawbacks, such as limited ink choices, the need for an ink-curing process, and lower conductivity caused by the use of dielectric binders [19]. A cutting-edge technique called laser scribing technology recently emerged as a promising alternative for fabricating customizable, graphene-like structures on a flexible plastic polyimide substrate [20,21]. This method offers ease of use, cost effectiveness, and does not require a mask. The resulting material, known as laser-scribed graphene (LSG), demonstrates remarkable characteristics such as tunable surface morphology, a large surface area, superior electrical characteristics, and mechanical resilience [22]. LSG-based electrodes (LSGEs) have extensive applications in electrocatalysis [23], supercapacitors [24], and electrochemical (bio)sensing [25,26,27,28], making it a highly attractive candidate to replace screen-printed electrodes (SPEs) as flexible-based sensor strips for glucose monitoring.

Numerous studies have investigated the use of various materials, including noble metals [29], alloys [30], transition metal oxides [31], and nanocomposites [32], as enzyme-free catalysts for electrocatalytic glucose oxidation. These materials demonstrated significantly enhanced catalytic activity compared to their bulk counterparts. For example, Gowthaman et al. introduced a glucose sensing platform by using copper nanostructures (CuNs) in combination with nitrogen-doped graphene (NG) on a glassy carbon electrode (GCE) [33]. Another study introduced a novel enzyme-free platform by depositing gold nanoparticles (Au NPs) onto a graphene and carbon nanotubes (CNTs) nanocomposite using supercritical fluid deposition [34]. Likewise, a recent publication showcased an enzyme-free glucose sensor using copper nanoparticles-based laser-scribed graphene (Cu NPs-LSG), which was created using a straightforward substrate-assisted electroless deposition (SAED) technique [35]. Furthermore, a glucose sensor utilizing copper oxide nanoparticles embedded in porous LSG (CuO NPs-LSG), prepared via focused sunlight crystalline CuO, demonstrated a detection range from 1 μM to 5 mM [36]. Although these enzyme-like nanomaterials exhibit effectiveness, one of their drawbacks is the requirement for highly alkaline conditions to achieve optimal catalytic activity. This limitation affects their practical use in certain fields, such as point-of-care diagnosis and on-site glucose monitoring [37].

On the other side, gold nanomaterials showed remarkable activity for glucose oxidation in both neutral and alkaline solutions [38]. As a result, there is increasing interest in utilizing gold nanoparticle-modified surfaces for enzyme-free electrochemical glucose sensors. The development of non-enzymatic glucose biosensors requires stringent criteria for electrocatalysis, interference rejection, and resistance to fouling, all of which depend heavily on the morphology and structure of gold nanomaterials on the electrodes [39]. Detecting glucose in neutral conditions can be challenging when using gold-based sensors due to the low concentration of OH- and Au(OH)ads species on the electrode surface. Nevertheless, employing an enzyme-free glucose sensor in a neutral medium offers significant advantages, as it eliminates the need for pH adjustment of the glucose-containing solution prior to analysis [40].

This work presents a novel approach where gold nanostructures (AuNs) are introduced as highly efficient electrocatalysts for glucose oxidation at neutral pH. These nanostructures are electrodeposited onto a laser-scribed graphene (LSG) electrode, which serves as a unique high 3D mesoporous graphene conductive support. The combination of the distinctive characteristics of the 3D porous LSGE and the excellent electrocatalytic activity of gold nanostructures results in the development of a highly sensitive glucose sensor with a wide linear range spanning from 0.5 to 20 mM at physiological pH, showcasing its broad applicability for glucose detection. Additionally, the study also looked into the effect of various major interferences on the glucose signal. Notably, our findings demonstrated that incorporating a Nafion^®^ film significantly enhanced both the stability and selectivity of the AuNs-LSGE sensor. This crucial improvement positions the developed sensor as an ideal choice for disposable sensor strips dedicated to glucose monitoring in human body fluids.

## 2. Materials and Methods

### 2.1. Chemicals and Reagents

The following chemicals were obtained from Sigma Aldrich (USA): D-(+)-glucose (C_6_H_12_O_6_, 99.5%), sulfuric acid (H_2_SO_4_, 98%), and gold (III) chloride hydrate (HAuCl_4_·3H_2_O, 99.9%). The Nafion™ perfluorinated resin solution was also purchased from Sigma Aldrich (USA). For the electrochemical oxidation of glucose, a neutral pH solution of 0.1 M phosphate-buffered saline (PBS) with a pH of 7.4 was prepared. The PBS solution was made by dissolving disodium hydrogen phosphate (Na_2_HPO_4_) and monopotassium phosphate (KH_2_PO_4_) in distilled water, which was obtained from SolvAchim (Casablanca, Morocco). A stock solution of 100 mM glucose was prepared in the 0.1 M phosphate buffer solution (PBS) and stored at 4 °C.

### 2.2. Apparatus and Electro-Analytical Techniques

Cyclic voltammetry (CV), linear sweep voltammetry (LSV), and amperometric measurements were conducted using a PalmSens BV 3 electrochemical instrument (Houten, The Netherlands), which was connected to a computer and operated through PSTrace 5.9 software. The morphology of the electrode surface was examined using scanning electron microscopy (Quattro ESEM) coupled with energy-dispersive X-ray spectroscopy (EDX) at National Center for Scientific and Technical Research (CNRST, Rabat, Morocco). To evaluate the electrochemical performance of the developed laser-scribed graphene electrode (LSGE) sensor, comparisons were made with commercially available screen-printed graphite electrodes (SPCEs) obtained from Metrohm Dropsens (Oviedo, Spain). All measurements were carried out at laboratory temperature, approximately 25 °C.

### 2.3. Fabrication of Laser-Scribed Graphene Electrode (LSGE)

The laser-scribed graphene electrode (LSGE) used in this study was kindly provided by Professor Khaled Nabil Salama at King Abdullah University of Science and Technology (Sensors Lab, Advanced Membranes and Porous Materials Center, KAUST, Saudi Arabia). The fabrication process of the LSGE was described in detail in our previous publications [41,42].

**Figure 1:**
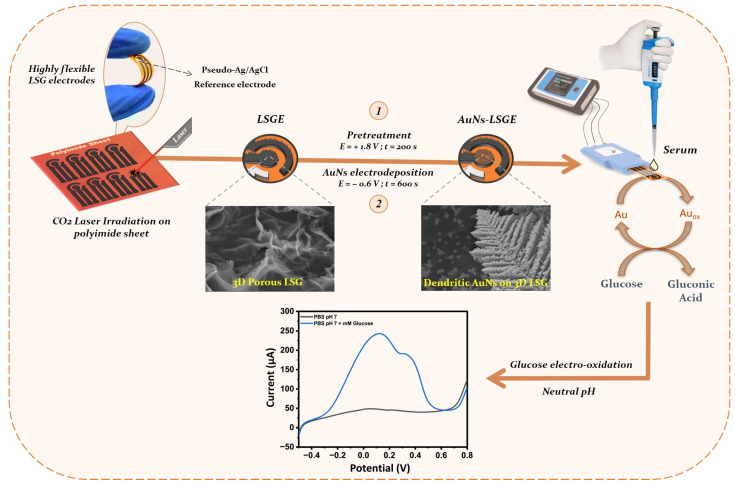
Schematic illustration of the preparation procedure of LSGE and AuNs-LSGE sensor, along with SEM images of the electrode surfaces and glucose electro-oxidation reaction for the CV measurements performed in 0.1 M PBS (pH 7.4).

Briefly, a commercially available polyimide film was utilized as a flexible substrate to create a three-electrode sensor system measuring 2.8 cm × 1.2 cm. The irradiation process was performed using a CO_2_ laser (Universal Laser Systems PLS6.75) with a wavelength of 10.6 µm and a spot size of 150 µm. The design of the LSGE involved miniaturizing the three-electrode system on the polyimide sheet, with a working electrode diameter of 3 mm (Figure 1). After laser scribing the electrodes, a layer of nail polish was applied to isolate the detection region and passivate the connection areas. A silver paste was applied on the reference electrode area and was electrochemically chlorinated in 1 M KCl at 1 V (vs. 3 M KCl Ag/AgCl commercial reference electrode) for 60 s to establish a pseudo-Ag/AgCl reference electrode. Cyclic voltammetry experiment was performed on LSGE with a concentration of 5 mM ferricyanides redox and 10 mM glucose at AuNs-LSGE (Figure S1, Supplementary Materials), and a stable electrochemical performance was observed. There was a minor variation in the potential window between the pseudo-Ag/AgCl reference electrode and the commercial Ag/AgCl electrode. 

### 2.4. Electrodeposition of Au Nanostructures on LSGE 

Before modifying the electrode, an external Ag/AgCl reference electrode (3 M KCl, +0.197 V vs. SHE) was used. The laser-scribed graphene electrode (LSGE) underwent pretreatment in 0.1 M PBS by applying a potential of +1.8 V for 200 s. This process effectively cleans and activates the electrode’s surface, eliminating impurities and creating an ideal foundation for subsequent electrodeposition processes. Additionally, the applied potential-induced electrochemical reactions bring about modifications to the graphene surface, potentially generating oxygen-rich functional groups and surface defects. These functional groups play a crucial role in enhancing the electrodeposition process by providing active sites for gold ions to bind to the graphene surface. This facilitates the formation of a uniform and strongly adhered gold coating [43]. The electrodeposition of the Au electrocatalyst on the LSG electrode surface was performed via potensiostatic mode conducted at a fixed deposition potential of −0.6 V for 600 s in a solution of 0.1 M H_2_SO_4_ containing the Au precursor (Figure S2). Subsequently, the electrodeposited gold on the LSGE was subjected to cyclic voltammetry in the range of −0.5 to +1.5 V (vs. 3 M KCl Ag/AgCl) at a scan rate of 100 mV·s^−1^ in 0.5 M H_2_SO_4_ for five consecutive cycles. This electrochemical polarization step facilitated the formation of an oxide-rich surface, increased the electro-active surface area, and promoted the formation of surface defects, clusters of adatoms, and atoms reordering. The same procedure was followed to prepare the AuNs electrocatalyst on the surface of the commercial screen-printed graphite electrode (SPCE).

### 2.5. Charcterization of Electrocatalytic Activity of AuNs-LSGE

The electrocatalytic activity of AuNs-LSG electrodes was evaluated through cyclic voltammetry (CV) and linear sweep voltammetry (LSV) measurements within a fixed potential range. These measurements were performed in 0.1 M PBS at pH 7.4, using a step potential of 5 mV and a scan rate of 50 mV·s^−1^, with the addition of an appropriate amount of glucose. Amperometric measurements were also performed in a stirred solution of 0.1 M PBS (pH 7.4) in a 5 mL electrochemical cell, with a stirring speed of 300 rpm. A selected applied potential was applied, and once a stable baseline current was achieved, glucose was added sequentially, and the resulting current responses were recorded. Additionally, chronoamperometric measurements were carried out for direct glucose analysis at an applied potential of +0.2 V vs. a pseudo-Ag/AgCl reference electrode. All measurements were conducted at ambient temperature with three replicates.

### 2.6. Real Sample Preparation

Human blood serum samples were obtained from two volunteers through a local clinical laboratory. To prepare the samples for analysis, 1 mL of serum was mixed with 1 mL of 0.1 M PBS solution containing 20% *v/v* ethanol, which served as a protein precipitating agent. The mixture was vigorously vortexed for 1 min to facilitate protein precipitation. Following this, the precipitated proteins were separated by centrifugation at 12,000 rpm for 5 min. The resulting clear supernatant was collected, which represented the protein-free human serum. To ensure further purification and removal of any remaining macromolecules, the protein-free serum was filtered using a 0.45 µm Millipore syringe filter. For the analysis, a 100 μL volume of the purified protein-free serum sample was directly applied onto the electrode surface.

## 3. Results and Discussion

### 3.1. Morphological and Structural Characterization of the Electrode Surface

**Figure 2:**
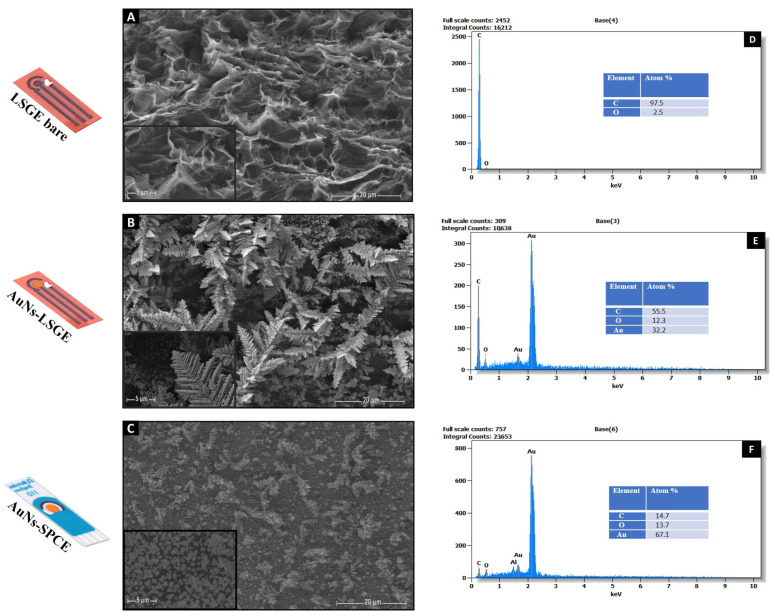
Scanning electron microscopy (SEM) images of (**A**) LSG electrode, (**B**) AuNs-LSG electrode, and (**C**) AuNs-SPCE. Scale bar for SEM images 20 μm and 5 μm (Insets). EDS spectra of (**D**) LSG electrode, (**E**) AuNs-LSG electrode, and (**F**) AuNs-SPCE.

Figure 2 displays the optical, SEM images, and X-ray energy dispersive spectroscopy (EDS) analysis of the LSG, AuNs-LSG, and AuNs-SPC electrodes. The SEM image depicted in Figure 2A clearly reveals that subjecting the polyimide (PI) sheet to CO_2_ laser treatment led to the formation of a highly interconnected 3D porous graphene structure in the LSGE, aligning with earlier studies. Upon electrochemically depositing gold on the LSG working electrode surface (Figure 2B), a noticeable coating of gold nanostructures (AuNs) was observed on the LSG working electrode. Comparatively, when compared to the commercially prepared SPCE under the same conditions (Figure 2C), the AuN anchored to the 3D LSG material exhibited unique interconnected dendrite gold nanostructures resembling feather branches that have proven to amplify the catalytic activity of the electrode. These distinct structures offer a larger surface area and improved access to catalytic active sites, thereby enhancing the electrochemical performance. In contrast, the SPCE featured a smooth, graphitic morphology with densely distributed spherical gold nanostructures. Consequently, the resulted morphology of the AuNs-LSG electrode exhibits significant potential for applications demanding efficient catalytic processes, as will be effectively demonstrated in the further section of this study.

The analysis of the EDS spectra revealed clear qualitative peaks, indicating the presence of elemental gold (Au), carbon (C), and oxygen (O). When examining the LSGE in its bare form (Figure 2D), the main peak was solely attributed to the carbonic nature of the graphene issued through laser scribing technology. Whereas, upon introducing gold through electrodeposition (Figure 2E), two distinct peaks emerged, signifying the existence of gold elements in different chemical states. This confirmed the successful coverage of the 3D porous LSG with gold nanostructures (AuNs). Interestingly, the atomic ratio of carbon to gold was determined to be 55% to 32%, indicating the presence of an alternate morphology of the deposited gold nanostructures on the 3D porous LSG. This observation was supported by the preservation of the porous features in the LSGE after modification, which was not the case for the dense morphology observed on the smooth SPCE surface in which the atomic ratio of carbon to gold on the SPCE was calculated to be 15% to 64% (Figure 2F), confirming the full coverage of the smooth surface with gold. Additionally, the significant presence of oxygen after the gold electrodeposition process on both AuNs-LSG and AuNs-SPC electrodes may be attributed to the formation of a gold oxide-rich surface during the electrochemical polarization step.

### 3.2. Electrocatatytic Activity of AuNs-LSGE toward Glucose Oxidation

**Figure 3:**
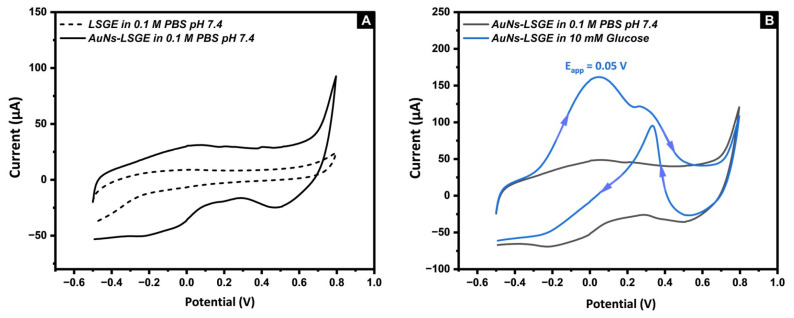
(**A**) CV in 0.1 M PBS pH 7.4 at bare LSGE (dashed line) and AuNs-LSGE (solid line), (**B**) CV in the absence (Black) and in the presence of 10 mM Glucose (Blue) in 0.1 M PBS pH 7.4, potential vs. pseudo-Ag/AgCl; scan rate: 50 mV·s^−1^.

The electrocatalytic properties of the developed AuNs-LSGE were studied using cyclic voltammetry in the absence and presence of glucose. Figure 3A shows CVs performed in 0.1 M PBS (pH of 7.4). In the absence of glucose, the AuNs-LSGE displayed the typical electrochemical behavior of gold including its oxidation and subsequent reduction of the gold oxides (solid line). Whereas, no catalytic behavior was noticed at the bare LSGE (dashed line). In addition, it was noted that the capacitive current density was greatly improved in the bare LSGE; this is mainly due to the higher surface area provided by the 3D networked structure of the LSG compared to the flat surface of the bare SPCE based on the previous SEM images. Upon the addition of 10 mM glucose (Figure 3B), CV responses of the AuNs-LSGE underwent significant changes and exhibited complex electrochemical behavior (blue line). The initial anodic peak observed during the forward scan at approximately 0.05 V was ascribed to the electrosorption of glucose, leading to the formation of an adsorbed intermediate and the subsequent release of a proton for each glucose molecule. At more positive potentials, the generation of OH(ads) species on the AuNs-LSGE surface promoted the catalyzed oxidation of the accumulated sub-products. Consequently, this released free Au active sites, enabling the direct oxidation of glucose into gluconolactone to take place at an approximate potential of 0.19 V, followed by its conversion to gluconate. During the reverse scan, the gold oxides formed in the forward cycle were reduced which resulted in a new and clean surface that can directly oxidize glucose, as observed at approximately 0.35 V. The decrease in current at positive potentials may be caused by the formation of Au oxide. This oxide competes with glucose for surface adsorption sites, hindering the direct electro-oxidation of glucose. A thorough mechanistic explanation of glucose oxidation on gold surfaces can be found in the literature [44,45].

**Figure 4:**
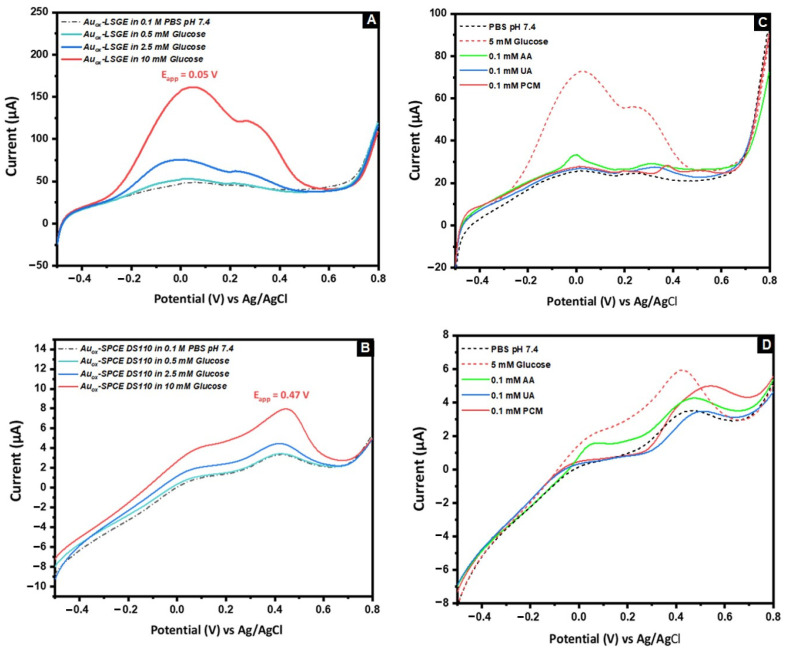
LSV of Glucose with different concentrations at (**A**) AuNs-LSGE and (**B**) AuNs-SPCE, LSV of 5 mM Glucose, 0.1 mM of AA, PCM, and UA at (**C**) AuNs-LSGE and (**D**) AuNs-SPCE; supporting electrolyte was 0.1 M PBS pH 7.4, potential vs. pseudo-Ag/AgCl; scan rate: 50 mV·s^−1^.

The developed sensor’s analytical characteristics were tested by comparing the results of the linear sweep voltammetry (LSV) response of various glucose concentrations (0.5, 2.5, and 10 mM) that are commonly found in biological matrices with a commercial SPCE prepared under the same electrodeposition conditions (Figure 4A,B). The results obtained suggest that the AuNs electrocatalyst incorporated on the 3D porous structure of the LSGE offers high sensitivity at lower overpotentials (the onset potential starts at nearly −0.3 V), allowing a direct dependence between the glucose concentration and the oxidation peak current. In addition, it is able to detect glucose concentrations as low as 0.5 mM, in contrast to the AuNs-SPCE where its sensitive current responses require higher potentials that are attributed to the second anodic peak located at +0.47 V. It was found that the detection currents at the AuNs-LSGE were higher than those at the AuNs-SPCE. The sensor’s high sensitivity is attributed to the large number of electro-active sites provided by the unique 3D porous structure of the LSG material, so it can be fully exploited by the deposited Au nanostructures. In comparison, the graphite-based SPE surface has fewer accessible sites for the detection reaction as it has a more compact and less porous structure. These results clearly indicate that the type of carbon support can greatly impact the glucose sensing performance of the electrodes.

A major difficulty in detecting glucose without enzymes is the presence of other substances that naturally occur in body fluids, such as ascorbic acid (AA), uric acid (UA), and paracetamol (PCM). These substances also undergo oxidation along with glucose. For this regard, a preliminary study was conducted to investigate the electro-oxidation potential of the tested interferences. As shown in Figure 4C, only AA has the lowest oxidation potential, making it the most likely to cause significant interference in the amperometric detection of glucose using the AuNs-LSGE, compared to PCM and UA which have higher anodic potentials. However, this is not the case with the AuNs-SPCE (Figure 4D), where all the tested substances strongly interfere with the sensitive anodic potential located at +0.45 V. Therefore, this study demonstrated the effectiveness of AuNs-LSGEs toward glucose electro-oxidation and stands out as a promising candidate for further analytical investigation as an enzyme-free glucose sensor in a neutral medium.

### 3.3. The Effect of the Applied Voltage and the Precursor Concentration on the Electrocatalytic Activity of AuNs-LSGE toward Glucose in Neutral pH

#### 3.3.1. The Effect of the Applied Voltage

**Figure 5:**
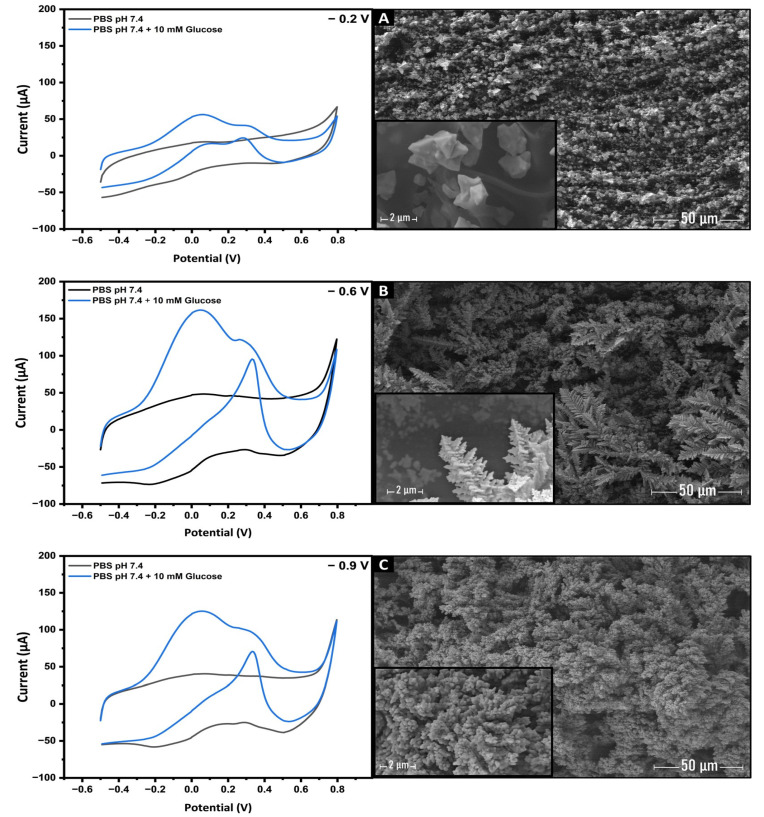
CVs recorded in 0.1 M PBS (pH 7.4) without (Black line) and with (Blue line) 10 mM of glucose with AuNs-LSGE after Au electrodeposition at fixed potentials of −0.2 (**A**), −0.6 (**B**), and −0.9 V (**C**); scan rate: 50 mV·s^−1^. SEM images: evolution of the gold nanostructures shape from tetrahedra-like morphology to feather-like branches morphology and eventually to bulk cauliflower-like Au nanostructures.

The choice of applied potential during the initial stage of electrodeposition plays a crucial role in determining the catalytic activity of the deposited gold nanostructures for glucose electro-oxidation. In Figure 5, the linear scanning voltammetry (LSV) of the AuNs-LSG electrodes, prepared under different applied potentials (−0.2, −0.6, and −0.9 V on a 50 mM HAuCl_4_ solution for 600 s), were recorded in a 0.1 M PBS solution containing 10 mM glucose. Notably, it was found that the electrodeposition potential of −0.6 V exhibited the highest faradaic current toward glucose oxidation.

To better understand how this parameter affects the growth and shape of deposited gold nanostructures, SEM analysis was conducted on the AuNs-LSG electrode surface. At an applied potential of −0.2 V (Figure 5A), the electrodeposited gold nanostructures exhibited a distinct morphology characterized by well-dispersed and uniformly shaped microstructures. Closer examination of the SEM image unveiled that these microstructures comprised tetrahedral-shaped nanoparticles. Subsequently, at an applied potential of −0.6 V (Figure 5B), a noticeable change in the morphology of the gold nanostructures was observed. The previously observed tetrahedral structures at −0.2 V were no longer visible. Instead, the nanostructures exhibited a denser and thicker appearance, resembling a Christmas-tree-like shape rooted from the conductive 3D networked substrate of LSG. In contrast, reducing the applied potential to −0.9 V led to the formation of dense and bulky flower-like structures (Figure 5C) that completely covered the surface of the LSG electrode. These structures exhibited a lower faradaic oxidation current compared to the morphology achieved at −0.6 V. This difference in electrocatalytic activity suggests that the morphology obtained at −0.6 V possessed highly favorable catalytic active sites for glucose electro-oxidation, leading to a significant increase in the faradaic current. On the other hand, the massive cauliflower-like structures formed at −0.9 V likely hindered the accessibility and availability of these catalytic active sites, resulting in a reduced faradaic oxidation current toward glucose.

These findings unequivocally demonstrate the significant influence of the electrodeposition potential on the morphology of gold nanostructures. Based on the results obtained, an electrodeposition potential of −0.6 V was determined as the optimal condition for subsequent experiments.

#### 3.3.2. Effect of HAuCl_4_ Precursor Concentration

The concentration of the gold precursor solution also plays a crucial role in determining the catalytic activities of metallic electrocatalysts. Consequently, gold nanostructures (AuNs) were electrodeposited using different concentrations of the gold precursor, HAuCl_4_. These modified nanostructured sensors (AuNs-LSGE) were subsequently examined for glucose electro-oxidation using CV.

**Figure 6:**
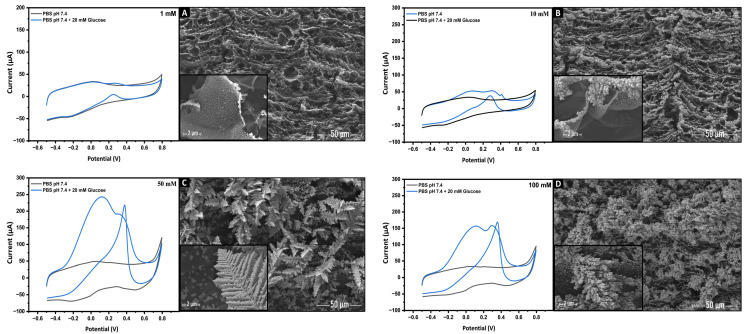
CVs recorded in 0.1 M PBS (pH 7.4) without (Black line) and with (Blue line) 10 mM of glucose with AuNs-LSGE after Au electrodeposition at a precursor concentration of 1 (**A**), 10 (**B**), 50 (**C**), and 100 mM (**D**). SEM images: evolution of the gold nanostructures shape from microclusters morphology to feather-like branches and eventually to bulk dendrite-like Au nanostructures.

Figure 6 presents the results of SEM characterization, which aimed to investigate the nucleation and morphological evolution of deposited AuNs. It was found that the morphology of the gold nanostructures underwent significant alterations in response to varying precursor concentrations during the electrodeposition process. As the precursor concentration increased, the gold structures evolved from small microclusters to denser and bulkier dendritic formations. At a gold precursor concentration of 1 mM (Figure 6A), the initial nucleation of gold occurred through the formation of small seed clusters dispersed on the graphene layers of the LSG electrode. However, this concentration proved insufficient for the electrocatalysis of glucose oxidation, as demonstrated in the cyclic voltammetry (CV). Subsequently, when the concentration was raised to 10 Mm (Figure 6B), the nucleation density significantly increased, leading to a greater abundance of small gold nuclei along the edges of the graphene layers on the LSG substrate.

Interestingly, when the concentration was further increased to 50 mM (Figure 6C), the nuclei grew and aggregated, resulting in well-oriented and interconnected dendrite-like gold nanostructures resembling feather branches. It was found that these nanostructures exhibited a higher electrocatalytic activity, as evidenced by the high faradaic current observed in CV during glucose electro-oxidation. The improved catalytic activity was mainly attributed to the higher density of catalytic active sites provided by this particular form of gold nanostructures. Upon reaching a concentration of 100 mM (Figure 6D), the aforementioned morphology continued to expand, resulting in the formation of more dense and bulky dendritic structures. However, the electrocatalytic glucose oxidation at this concentration exhibited relatively lower efficiency compared to that observed at 50 mM. This diminished performance can be ascribed to the reduction in the electrocatalytic surface area, which usually occurs as the size of the nanostructures increases.

The findings of this study clearly indicate the pivotal role of the precursor solution concentration in governing the morphological characteristics of gold nanostructures which significantly affects the presence of more catalytic sites as it is advantageous for facilitating the electrocatalytic oxidation of glucose molecules. Based on these results, 50 mM was chosen as the optimal gold precursor concentration for the subsequent experment.

### 3.4. Electro-Analytical Performance of AuNs-LSGE Sensor

After preparation of the AuNs-LSGE sensor, the electrochemical detection of glucose was carried out using the linear sweep voltammetry and constant potential amperometric techniques. All experiments were performed in a neutral PBS solution at physiological pH level (pH 7.4). A concentration range of 0.5 mM to 20.0 mM was achieved, which encompasses both physiological and elevated levels of glucose in a wide range of samples.

#### 3.4.1. Voltammetric Detection

The performance of the glucose sensor based on the AuNs-LSGE was assessed using linear sweep voltammetry (LSV). The voltage was scanned from −0.4 V to +0.8 V at a scan rate of 50 mV·s^−1^. Figure S2 demonstrates the LSV curves obtained for the detection of glucose using the AuNs-LSGE sensor. A gradual increase in the current of the first anodic peak was observed as the glucose concentration increased from 0.5 to 20 mM. The relationship between the variation in peak current (during LSV of glucose solutions) and the concentrations of glucose is depicted. A linear range was identified, with a detection limit of 0.11 mM. The fitted linear regression equation is ∆I (µA) = 8.28 [C(mM)] + 6.43 (R^2^ = 0.991). The sensitivity for the detection of glucose was 112 µM.

#### 3.4.2. Amperometric Detection

**Figure 7:**
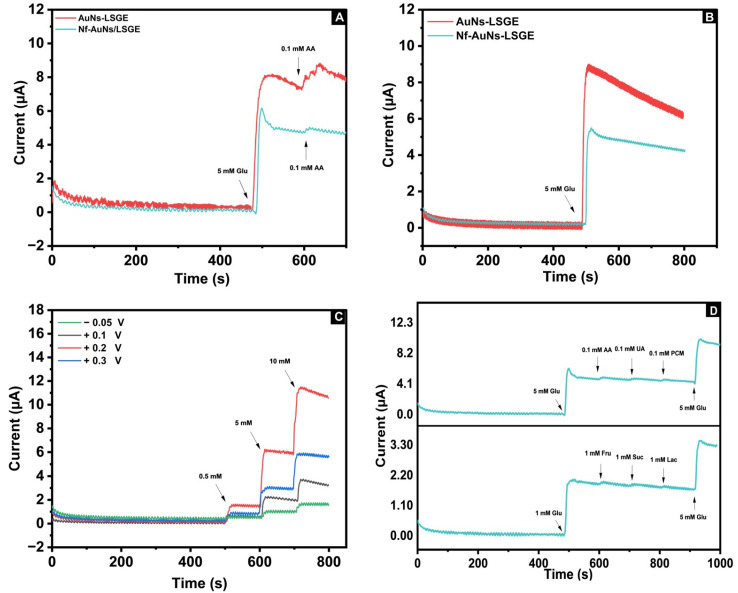
(**A**) I (µA) vs. t (s) curve of 5 mM glucose, 0.1 mM AA at AuNs-LSGE and at Nafion-AuNs-LSGE; (**B**) I (µA) vs. t (s) curve of 5 mM glucose at AuNs-LSGE and Nafion-AuNs-LSGE; (**C**) I (µA) vs. t (s) of glucose at Nafion-AuNs-LSGE with increasing concentrations (0.5, 5, and 10 mM) of glucose at different applied potentials; and (**D**) I (µA) vs. t (s) of 5 mM glucose at Nafion-AuNs-LSGE in the presence of biological compounds (0.1 mM AA, 0.1 mM UA, and 0.1 mM PCM) and sugar compounds (1 mM fructose, 1 mM sucrose, and 1 mM lactose). Applied potential: +0.2 V vs. pseudo-Ag/AgCl; supporting electrolyte is 0.1 M PBS pH 7.4.

In order to evaluate the analytical usefulness of the developed AuNs-LSGE sensor, the amperometric method (current vs. time) is more desirable than the voltammetric one due to its high sensitivity, real-time monitoring, and practical applications. According to the previous CV results, one of the major obstacles in non-enzymatic glucose sensing is the interference of ascorbic acid (AA) on the electrochemical signals. AA oxidation occurs at a low potential value, making it highly likely to interfere with the amperometric detection of glucose using the AuNs-LSGE, as demonstrated in Figure 7A. It was found that this issue was addressed by employing a Nafion membrane, a widely known cross-linked polymer with distinct characteristics when in contact with an aqueous solution, and it features polymeric domains measuring tens of nanometers in size, along with interconnected pores and channels. Thanks to these pores and channels, glucose can effectively permeate through the Nafion membrane. Thus, coating the electrode surface with 2 µL of Nafion membrane (5%) using the drop-casting method significantly reduced the amperometric signal of the ascorbic acid (AA), since it can act as a semi-permeable barrier for several anionic substances due to its negatively charged functional groups, allowing it to repel anionic species, as in the case of ascorbic acid.

Figure 7B shows the amperometric responses toward glucose oxidation upon the addition of 5 mM of glucose at a working potential of +0.2 V. As it can be seen, the AuNs-LSGE exhibits a high current response that reaches dynamic equilibrium in less than three seconds, demonstrating that the AuNs-LSG electrode provides a very sensitive and fast amperometric response. However, remarkable signal noise was noticed with a continuous decrease in current response when the sensor was monitored within a long period of time. The mentioned concern may arise from the significant adsorption of byproducts resulting from the oxidation of glucose onto the large surface area provided by the 3D porous AuNs-LSG support materials. This process creates a barrier that hinders the entry of glucose molecules to the catalytic active sites, thereby directly affecting the lifespan of the response exhibited by the AuNs electrocatalyst.

##### Applied Amperometric Potential

As it is well known, the selection of the applied potential is also an important factor to obtain a highly sensitive and selective anodic amperometric response in the electro-oxidation of glucose. Figure 7C shows the amperometric responses of the Nf-AuNs-LSGE at various anodic potentials ranging from −0.05 to +0.3 V with a continuous increase in glucose concentration to the stirred phosphate buffered solution. At potentials of −0.05 and 0.1 V, the current increased slightly and reached constant values, but the sensitivity was not good enough to offer a wide linear range if the glucose concentration continued to increase, due to the incomplete oxidation of glucose at low potentials, thereby blocking some active sites. Whereas, at +0.2 V, the current response was highly sensitive than that observed at +0.3 V, where the amperometric response was stable, but the sensitivity was reduced which might be attributed to the adsorption of glucose intermediates onto the electrode surface. As a result, a potential of +0.2 V was selected as the optimal value. Thus, by simply applying this potential value, high selectivity can be achieved in the presence of some common oxidizable compounds found in biological fluids, such as uric acid and paracetamol. This is in line with the previous CV results.

##### Interference Study

As stated previously, a significant obstacle in non-enzymatic glucose sensors is the interference of electrochemical signals arising from easily oxidizable organic substances such as ascorbic acid (AA), uric acid (UA), and paracetamol (PCM), as well as some other carbohydrates such as sucrose (Suc), lactose (Lac), and fructose (Fru).

The typical range for glucose levels in the body is 3–8 mM, significantly higher compared to interfering species such as AA (approximately 0.1 mM), PCM (approximately 0.1 mM), and UA (approximately 0.02 mM). The results shown in Figure 7D demonstrate that the oxidation current of the interfering substances being studied was negligible in comparison to the observed current for 5 mM glucose. This can be mainly attributed to two factors. Firstly, the applied voltage of +0.2 V significantly decreases the anodic current response of certain interfering species that undergo oxidation at higher potentials. Secondly, the incorporation of the Nafion membrane onto the surface of the AuNs-LSGE, which consists of negatively charged functional groups, acts as a partially permeable barrier and effectively repels anionic substances, such as ascorbic acid and uric acid, particularly at neutral pH. Additionally, the sensor did not exhibit any measurable response to lactose, fructose, and sucrose. Indeed, the specificity for glucose in comparison to the other tested structurally related sugars was possibly attributed to the stereochemistry involved in different sugars. The orientation of the carbon C1-H in cyclic glucose plays a crucial role, and steric effects may affect the kinetics of the oxidation pathway in other sugars, resulting in a lower response observed in other sugars studied [46,47].

Therefore, the developed electrode has the potential to diminish the influence of the interference species, making it a highly effective tool for accurately detecting glucose in clinical diagnosis and food control applications as well.

##### Sensitivity

**Figure 8:**
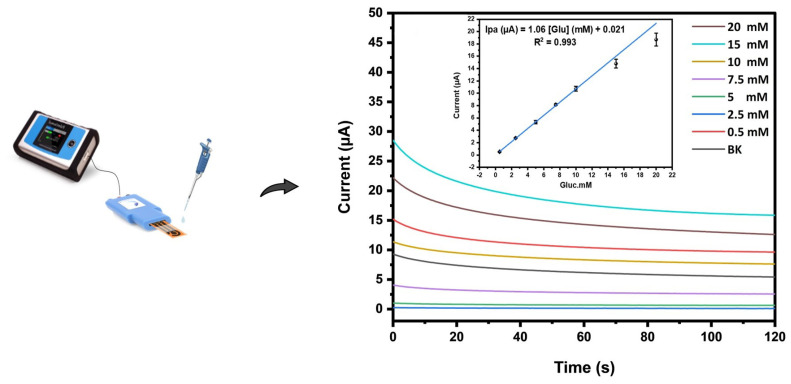
Chronoamperometry (CA) of glucose with different concentrations (0.5, 2.5, 5, 10, 15, and 20 mM) at Nf-AuNs-LSGE. Inset represents the corresponding calibration curve. Supporting electrolyte is 0.1 M PBS pH 7.4; applied potential: +0.2 V vs. pseudo-Ag/AgCl.

In order to ensure its portability, the proposed sensing platform underwent testing in a disposable mode using the chronoamperometric technique. The tests were conducted in PBS pH 7.4 under the previously optimized conditions. Various glucose concentrations were dropped to the Nf-AuNs-LSGE strip, with an applied potential of +0.2 V (Figure 8). It was observed that the current increased as the glucose concentration increased. The inset of the corresponding figure illustrates the relationship between the current recorded at 60 s and the corresponding glucose concentration. The results demonstrated a wider linear range spanning from 0.5 to 20 mM, with a correlation coefficient of R^2^ = 0.993. The sensitivity of the sensor was calculated to be 1.06 ± 0.05 μA mM^−1^, and the detection limit was estimated to be 0.21 mM based on Equation (1), where σ was the standard deviation of three replicates (*n* = 3), and S was the slope of the calibration curve.

LOD = 3σ/S(1)

Importantly, the upper limit of the linear range far exceeded the physiological glucose levels typically observed (ranging from 3 to 8 mM). This suggests that the developed sensor would prove valuable in detecting glucose concentrations within both normal and abnormal glucose levels.

The results indicate that the designed sensor has the potential to accurately monitor glucose levels with high sensitivity without the need for complicated preparation of electrocatalyst nanomaterials such as binary metallic nanomaterials or conducting polymers-based nanocomposites, as outlined in Table 1.

##### Life Time and Reproducibility Assays

The shelf-life of the sensor was monitored by recording the amperometric response of the Nf-AuNs-LSGE to 5 mM glucose after it was stored in a dry state for various periods of time at an ambient temperature (Figure S4). The results show that the amperometric current was reduced by 4%, 7%, and 14% of its original response after 5, 10, and 20 days, respectively.

For the purpose of studying reproducibility, a set of consecutive measurements was carried out on 5 mM glucose. The results demonstrated a relative standard deviation (RSD) of 5.2% among six electrodes, all prepared under the same conditions. This finding highlights the good reliability of the measurements.

#### 3.4.3. Real Sample Analysis

To evaluate the applicability of the proposed glucose sensor for the analysis, as discussed in Section 2.6, the serum samples were treated to reduce the matrix effect that arises from the nonspecific adsorption of the macromolecules onto the electrode surface. A total of 100 µL of the pretreated serum samples was analyzed directly on the developed sensor based on the established calibration curve. Then, the recovery was tested by spiking 2.5 mM of glucose, as shown in Table 2. Furthermore, the results obtained from the developed glucose sensor were compared to those obtained from a commercial Glucometer. The measured values showed good agreement with the data recorded by the commercial device. This result underscores the high potential of the developed non-enzymatic glucose sensor for the precise analysis of glucose in real clinical samples.

## 4. Conclusions

In summary, a novel disposable glucose sensor was developed by combining the unique features of laser-scribed 3D graphene electrodes (LSGEs) with the excellent electrocatalytic activity of the electrodeposited gold nanostructures (AuNs) toward glucose oxidation at physiological medium (pH 7.4). The fabrication process of the LSGE and electrodeposition of AuNs was optimized and investigated. The AuNs act as an enzyme-like activity to catalyze glucose electro-oxidation, and the LSGEs ensure a high surface area of the sensing material. The introduction of Nafion^®^ film significantly improved both the stability and selectivity of the AuNs-LSGE sensor. The electro-analytical performances of the developed sensor offer a wide linear range from 0.5 to 20 mM, with a limit of detection of 0.21 mM. This sensing strip presents an affordable, highly sensitive, and selective non-enzymatic solution for detecting glucose, which can be useful for the self-monitoring of glucose levels and food management.

## Figures and Tables

**Table 1:** Analytical performances of non-enzymatic glucose electrochemical sensors based on metal/metallic nanocomposites.

Electrode Configuration	Applied Potential (V)	Linear Range (mM)	LOD (µM)	Medium	Ref.
Pd@Au@MoS_2_-GCE ^1^	−0.1	0.5–20	400	Alkaline	[48]
Pt@f-CNF-GCE ^2^	+0.51	1–10	0.42	Neutral	[46]
PtAu@C-GCE ^3^	+0.35	1–10	2	Neutral	[47]
Fe@Pt coreshell-GCE ^4^	−0.15	1–16	300	Neutral	[49]
PtNi@MWCNTs-GCE ^5^	+0.1	0.1–9	0.3	Neutral	[50]
Pd@Co@CNTs-GCE ^6^	+0.5	0.001–2.4	1	Alkaline	[51]
CoPNs-SPCE ^7^	+0.65	1–30	300	Neutral	[52]
Au@MPTS@Pt-SPCE ^8^	+0.4	1–18	2	Neutral	[53]
Pt@CuO@rGO-SPCE ^9^	+0.35	2–12	10	Alkaline	[54]
Pt-SPCE ^10^	+0.65	1–30	-	Neutral	[55]
CuONPs-LSGE ^11^	+0.4	0.001–5	0.1	Alkaline	[36]
CuNPs-LIG ^12^	+0.5	0.001–6	0.39	Alkaline	[56]
AuNi-LIG ^13^	+0.1	0–30	-	Alkaline	[57]
Nf-Au-LSGE	+0.2	0.5–20	210	Neutral	This work

Abbreviations: ^1^ molybdenum disulfide (MoS_2_) nanosheets supported Au-Pd bimetallic nanoparticles; ^2^ Pt Nanoparticles Decoration of Functionalized Carbon Nanofibers; ^3^ bimetallic PtAu/C-Based Nanocomposites; ^4^ Fe@Pt core–shell nanoparticles; ^5^ bimetallic PtNi-Multi-walled carbon NT nanocomposites; ^6^ palladium-cobalt nanoparticles supported over carbon nanotubes; ^7^ cobalt phosphate nanostructures (CPNs); ^8^ double-layer self-assembly of (3 mercaptopropyl)trimethoxysilane (MPTS) on a gold substrate and co-deposition of a platinum–copper alloy; ^9^ platinum nanocubes and copper oxide nanoflowers decorated reduced graphene oxide (rGO); ^10^ nanoporous Pt; ^11^ copper oxide nanoparticles (CuO NPs); ^12^ Cu nanoparticles anchored on laser-induced graphene (LIG); and ^13^ nickel and gold layer on (LIG) electrodes.

**Table 2:** Analytical application of the developed sensor for glucose detection in human blood serum (*n* = 5).

	[Glucose] Before Spiking (mM)	[Glucose] Added (mM)	[Glucose] Expected (mM)	[Glucose] Found by Commercial Glucometer ± SD (mM)	[Glucose] Found by Developed Sensor ± SD (mM)	Recovery%
**Human serum 1**	2.7 ± 0.1	2.5 mM	5.2	5.2 ± 0.1	5.3 ± 0.2	101.2
**Human serum 2**	3.3 ± 0.1	2.5 mM	5.8	5.7 ± 0.1	6.0 ± 0.25	103.4

## Data Availability

The data used to support the findings of this study are available from the corresponding author upon request.

## Supplementary Materials

The following are available online at https://www.mdpi.com/article/10.3390/bios13070678/s1. Figure S1: CV of 5 mM Ferricyanides redox at LSGE (**A**); and CV of 10 mM Glucose at AuNs-LSGE (**B**), with commercial Ag/AgCl RE (Black) and Pseudo-Ag/AgCl RE (Red), scan rate v = 50 mV·s^−1^. Figure S2: electrodeposition chronoamperograms of AuNs (**A**) at different potentials −0.2, −0.6, and −0.9 V vs. Ag/AgCl (3 M KCl) for 600 s in a 0.5 M H_2_SO_4_; (**B**) at different gold precursor concentrations 1, 10, 50, and 100 mM; and (**C**) five successive CV scans of AuNs-LSGE performed in 0.5 M H_2_SO_4_ solution, scan rate 100 mV s^−1^. Figure S3: (**A**) LSV of glucose with different concentrations (0.5, 2.5, 5, 10, and 20) at AuNs-LSGE in 0.1 M PBS pH 7.4; scan rate: 50 mV·s^−1^. (**B**) The corresponding calibration curve. Figure S4: Current response % of 5 mM glucose over days recorded from I vs. time (s).

## References

[1] Moore B., Zhou L., Rolland F., Hall Q., Cheng W.H., Liu Y.X., Hwang I., Jones T., Sheen J. (2003). Role of the Arabidopsis Glucose Sensor HXK1 in Nutrient, Light, and Hormonal Signaling. Science.

[2] Tian H., Yang Y., Xie D., Cui Y.L., Mi W.T., Zhang Y., Ren T.L. (2014). Wafer-Scale Integration of Graphene-Based Electronic, Optoelectronic and Electroacoustic Devices. Sci. Rep..

[3] Wang H.C., Lee A.R. (2015). Recent Developments in Blood Glucose Sensors. J. Food Drug Anal..

[4] Heller A., Feldman B. (2008). Electrochemical Glucose Sensors and Their Applications in Diabetes Management. Chem. Rev..

[5] Zhu L., She Z.G., Cheng X., Qin J.J., Zhang X.J., Cai J., Lei F., Wang H., Xie J., Wang W. (2020). Association of Blood Glucose Control and Outcomes in Patients with COVID-19 and Pre-Existing Type 2 Diabetes. Cell Metab..

[6] Zimmet P.Z., Magliano D.J., Herman W.H., Shaw J.E. (2014). Diabetes: A 21st Century Challenge. Lancet Diabetes Endocrinol..

[7] King E.J., Garner R.J. (1947). The Colorimetric Determination of Glucose. J. Clin. Pathol..

[8] Liu M., Liu R., Chen W. (2013). Graphene Wrapped Cu2O Nanocubes: Non-Enzymatic Electrochemical Sensors for the Detection of Glucose and Hydrogen Peroxide with Enhanced Stability. Biosens. Bioelectron..

[9] Attaallah R., Elfadil D., Amine A. (2021). Screening Study of Enzymatic Inhibition of Medicinal Plants for the Treatment of Diabetes Using a Glucometer Biosensor Approach and Optical Method. J. Herb. Med..

[10] Wang J. (2008). Electrochemical Glucose Biosensors. Chem. Rev..

[11] Kucherenko I.S., Soldatkin O.O., Dzyadevych S.V., Soldatkin A.P. (2020). Electrochemical Biosensors Based on Multienzyme Systems: Main Groups, Advantages and Limitations – A Review. Anal. Chim. Acta.

[12] Kim J., Campbell A.S., Wang J. (2018). Wearable Non-Invasive Epidermal Glucose Sensors: A Review. Talanta.

[13] Park S., Boo H., Chung T.D. (2006). Electrochemical Non-Enzymatic Glucose Sensors. Anal. Chim. Acta.

[14] Toghill K.E., Compton R.G. (2010). Electrochemical Non-Enzymatic Glucose Sensors: A Perspective and an Evaluation. Int. J. Electrochem. Sci..

[15] Hwang D.W., Lee S., Seo M., Chung T.D. (2018). Recent Advances in Electrochemical Non-Enzymatic Glucose Sensors—A Review. Anal. Chim. Acta.

[16] Zeng Y., Wang J., Wang Z., Chen G., Yu J., Li S., Li Q., Li H., Wen D., Gu Z. (2020). Colloidal Crystal Microneedle Patch for Glucose Monitoring. Nano Today.

[17] Wang J., Angnes L. (1992). Miniaturized Glucose Sensors Based on Electrochemical Codeposition of Rhodium and Glucose Oxidase onto Carbon-Fiber Electrodes. Anal. Chem..

[18] Vashist S.K., Zheng D., Al-Rubeaan K., Luong J.H.T., Sheu F.S. (2011). Technology behind Commercial Devices for Blood Glucose Monitoring in Diabetes Management: A Review. Anal. Chim. Acta.

[19] Ahmed M.U., Hossain M.M., Safavieh M., Wong Y.L., Rahman I.A., Zourob M., Tamiya E. (2015). Toward the Development of Smart and Low Cost Point-of-Care Biosensors Based on Screen Printed Electrodes. Crit. Rev. Biotechnol..

[20] Strong V., Dubin S., El-Kady M.F., Lech A., Wang Y., Weiller B.H., Kaner R.B. (2012). Patterning and Electronic Tuning of Laser Scribed Graphene for Flexible All-Carbon Devices. ACS Nano.

[21] Tian H., Mohammad M.A., Mi W.-T., Yang Y., Ren T.-L. (2016). Laser-Scribing Technology for Wafer-Scale Graphene Devices. Advances in Carbon Nanostructures.

[22] Tian H., Chen H.Y., Ren T.L., Li C., Xue Q.T., Mohammad M.A., Wu C., Yang Y., Wong H.S.P. (2014). Cost-Effective, Transfer-Free, Flexible Resistive Random Access Memory Using Laser-Scribed Reduced Graphene Oxide Patterning Technology. Nano Lett..

[23] Zhang J., Ren M., Wang L., Li Y., Yakobson B.I., Tour J.M. (2018). Oxidized Laser-Induced Graphene for Efficient Oxygen Electrocatalysis. Adv. Mater..

[24] Wen F., Hao C., Xiang J., Wang L., Hou H., Su Z., Hu W., Liu Z. (2014). Enhanced Laser Scribed Flexible Graphene-Based Micro-Supercapacitor Performance with Reduction of Carbon Nanotubes Diameter. Carbon N. Y..

[25] Ghanam A., Lahcen A.A., Beduk T., Alshareef H.N., Amine A., Salama K.N. (2020). Laser Scribed Graphene: A Novel Platform for Highly Sensitive Detection of Electroactive Biomolecules. Biosens. Bioelectron..

[26] Rauf S., Lahcen A.A., Aljedaibi A., Beduk T., Ilton de Oliveira Filho J., Salama K.N. (2021). Gold Nanostructured Laser-Scribed Graphene: A New Electrochemical Biosensing Platform for Potential Point-of-Care Testing of Disease Biomarkers. Biosens. Bioelectron..

[27] Lahcen A.A., Rauf S., Beduk T., Durmus C., Aljedaibi A., Timur S., Alshareef H.N., Amine A., Wolfbeis O.S., Salama K.N. (2020). Electrochemical Sensors and Biosensors Using Laser-Derived Graphene: A Comprehensive Review. Biosens. Bioelectron..

[28] Liu X., Cheng H., Zhao Y., Wang Y., Li F. (2022). Portable Electrochemical Biosensor Based on Laser-Induced Graphene and MnO_2_ Switch-Bridged DNA Signal Amplification for Sensitive Detection of Pesticide. Biosens. Bioelectron..

[29] Ghanam A., Haddour N., Mohammadi H., Amine A., Sabac A., Buret F. (2022). Nanoporous Cauliflower-like Pd-Loaded Functionalized Carbon Nanotubes as an Enzyme-Free Electrocatalyst for Glucose Sensing at Neutral PH: Mechanism Study. Sensors.

[30] Wang J., Gao H., Sun F., Xu C. (2014). Nanoporous PtAu Alloy as an Electrochemical Sensor for Glucose and Hydrogen Peroxide. Sens. Actuators B Chem..

[31] Niu X., Li X., Pan J., He Y., Qiu F., Yan Y. (2016). Recent Advances in Non-Enzymatic Electrochemical Glucose Sensors Based on Non-Precious Transition Metal Materials: Opportunities and Challenges. RSC Adv..

[32] Chen J., Zhang W.D., Ye J.S. (2008). Nonenzymatic Electrochemical Glucose Sensor Based on MnO2/MWNTs Nanocomposite. Electrochem. commun..

[33] Gowthaman N.S.K., Raj M.A., John S.A. (2017). Nitrogen-Doped Graphene as a Robust Scaffold for the Homogeneous Deposition of Copper Nanostructures: A Nonenzymatic Disposable Glucose Sensor. ACS Sustain. Chem. Eng..

[34] Wu J.W., Wang C.H., Wang Y.C., Chang J.K. (2013). Ionic-Liquid-Enhanced Glucose Sensing Ability of Non-Enzymatic Au/Graphene Electrodes Fabricated Using Supercritical CO2 Fluid. Biosens. Bioelectron..

[35] Lin S., Feng W., Miao X., Zhang X., Chen S., Chen Y., Wang W., Zhang Y. (2018). A Flexible and Highly Sensitive Nonenzymatic Glucose Sensor Based on DVD-Laser Scribed Graphene Substrate. Biosens. Bioelectron..

[36] Prabhakaran A., Nayak P. (2020). Surface Engineering of Laser-Scribed Graphene Sensor Enables Non-Enzymatic Glucose Detection in Human Body Fluids. ACS Appl. Nano Mater..

[37] Wang G., He X., Wang L., Gu A., Huang Y., Fang B., Geng B., Zhang X. (2012). Non-Enzymatic Electrochemical Sensing of Glucose. Microchim. Acta 2012 1803.

[38] Feng D., Wang F., Chen Z. (2009). Electrochemical Glucose Sensor Based on One-Step Construction of Gold Nanoparticle–Chitosan Composite Film. Sensors Actuators B Chem..

[39] Tee S.Y., Teng C.P., Ye E. (2017). Metal Nanostructures for Non-Enzymatic Glucose Sensing. Mater. Sci. Eng. C.

[40] Hassan M.H., Vyas C., Grieve B., Bartolo P. (2021). Recent Advances in Enzymatic and Non-Enzymatic Electrochemical Glucose Sensing. Sensors.

[41] Berni A., Ait lahcen A., Amine A. Electrochemical Sensing of Paracetamol Using 3D Porous Laser Scribed Graphene Platform. Electroanalysis.

[42] Berni A., Ait Lahcen A., Salama K.N., Amine A. (2022). 3D-Porous Laser-Scribed Graphene Decorated with Overoxidized Polypyrrole as an Electrochemical Sensing Platform for Dopamine. J. Electroanal. Chem..

[43] Beduk T., De Oliveira Filho J.I., Ait Lahcen A., Mani V., Salama K.N. (2021). Inherent Surface Activation of Laser-Scribed Graphene Decorated with Au and Ag Nanoparticles: Simultaneous Electrochemical Behavior toward Uric Acid and Dopamine. Langmuir.

[44] Tominaga M., Shimazoe T., Nagashima M., Taniguchi I. (2005). Electrocatalytic Oxidation of Glucose at Gold Nanoparticle-Modified Carbon Electrodes in Alkaline and Neutral Solutions. Electrochem. commun..

[45] Li Y., Song Y.Y., Yang C., Xia X.H. (2007). Hydrogen Bubble Dynamic Template Synthesis of Porous Gold for Nonenzymatic Electrochemical Detection of Glucose. Electrochem. commun..

[46] Singh B., Dempsey E., Dickinson C., Laffir F. (2012). Inside/Outside Pt Nanoparticles Decoration of Functionalised Carbon Nanofibers (Pt19.2/f-CNF80.8) for Sensitive Non-Enzymatic Electrochemical Glucose Detection. Analyst.

[47] Singh B., Laffir F., McCormac T., Dempsey E. (2010). PtAu/C Based Bimetallic Nanocomposites for Non-Enzymatic Electrochemical Glucose Detection. Sensors Actuators B Chem..

[48] Li X., Du X. (2017). Molybdenum Disulfide Nanosheets Supported Au-Pd Bimetallic Nanoparticles for Non-Enzymatic Electrochemical Sensing of Hydrogen Peroxide and Glucose. Sens. Actuators B Chem..

[49] Mei H., Wu W., Yu B., Wu H., Wang S., Xia Q. (2016). Nonenzymatic Electrochemical Sensor Based on Fe@Pt Core–Shell Nanoparticles for Hydrogen Peroxide, Glucose and Formaldehyde. Sens. Actuators B Chem..

[50] Mei H., Wu H., Wu W., Wang S., Xia Q. (2015). Ultrasensitive Electrochemical Assay of Hydrogen Peroxide and Glucose Based on PtNi Alloy Decorated MWCNTs. RSC Adv..

[51] Huang B., Wang Y., Lu Z., Du H., Ye J. (2017). One Pot Synthesis of Palladium-Cobalt Nanoparticles over Carbon Nanotubes as a Sensitive Non-Enzymatic Sensor for Glucose and Hydrogen Peroxide Detection. Sens. Actuators B Chem..

[52] Tomanin P.P., Cherepanov P.V., Besford Q.A., Christofferson A.J., Amodio A., McConville C.F., Yarovsky I., Caruso F., Cavalieri F. (2018). Cobalt Phosphate Nanostructures for Non-Enzymatic Glucose Sensing at Physiological PH. ACS Appl. Mater. Interfaces.

[53] McCormick W., McDonagh P., Doran J., McCrudden D. (2021). Covalent Immobilisation of a Nanoporous Platinum Film onto a Gold Screen-Printed Electrode for Highly Stable and Selective Non-Enzymatic Glucose Sensing. Catalysts.

[54] Dhara K., Stanley J., Ramachandran T., Nair B.G., Satheesh S.B. (2014). Pt-CuO Nanoparticles Decorated Reduced Graphene Oxide for the Fabrication of Highly Sensitive Non-Enzymatic Disposable Glucose Sensor. Sens. Actuators B Chem..

[55] Lee S., Lee J., Park S., Boo H., Kim H.C., Chung T.D. (2018). Disposable Non-Enzymatic Blood Glucose Sensing Strip Based on Nanoporous Platinum Particles. Appl. Mater. Today.

[56] Zhang Y., Li N., Xiang Y., Wang D., Zhang P., Wang Y., Lu S., Xu R., Zhao J. (2020). A Flexible Non-Enzymatic Glucose Sensor Based on Copper Nanoparticles Anchored on Laser-Induced Graphene. Carbon N. Y..

[57] Zhu J., Liu S., Hu Z., Zhang X., Yi N., Tang K., Dexheimer M.G., Lian X., Wang Q., Yang J. (2021). Laser-Induced Graphene Non-Enzymatic Glucose Sensors for on-Body Measurements. Biosens. Bioelectron..

