# Supplementary material for: Feather-like Gold Nanostructures Anchored onto 3D Mesoporous Laser-Scribed Graphene: A Highly Sensitive Platform for Enzymeless Glucose Electrochemical Detection in Neutral Media
Source: Biosensors (Basel). 2023 Jun 25;13(7):678. doi: 10.3390/bios13070678 (PMC10377420; doi:10.3390/bios13070678)
Supplement: Supplementary file 1 [file biosensors-13-00678-s001.zip › biosensors-2441615-supplementary.pdf]

biosensors

Supplementary Materials

Feather-like Gold Nanostructures Anchored onto 3D Mesoporous Laser-Scribed Graphene: A Highly Sensitive Platform for Enzymeless Glucose Electrochemical Detection in Neutral Media

Achraf Berni [1,2], Aziz Amine [1,*], Juan José García-Guzmán [2], Laura Cubillana-Aguilera [2] and José María Palacios-Santander [2]

1 Laboratory of Process Engineering and Environment, Faculty of Sciences and Techniques, Hassan II University of Casablanca, P.A. 149, Mohammedia 28810, Morocco; achrafberni@gmail.com
2 Department of Analytical Chemistry, Institute of Research on Electron Microscopy and Materials (IMEYMAT), Faculty of Sciences, Campus de Excelencia Internacional del Mar (CEIMAR), University of Cadiz, Campus Universitario de Puerto Real, Polígono del Río San Pedro S/N, 11510 Puerto Real, Cádiz, Spain; juanjogg91@gmail.com (J.J.G.-G.); laura.cubillana@uca.es (L.C.-A.); josem.palacios@uca.es (J.M.P.-S.)
* Correspondence: aziz.amine@fstm.ac.ma

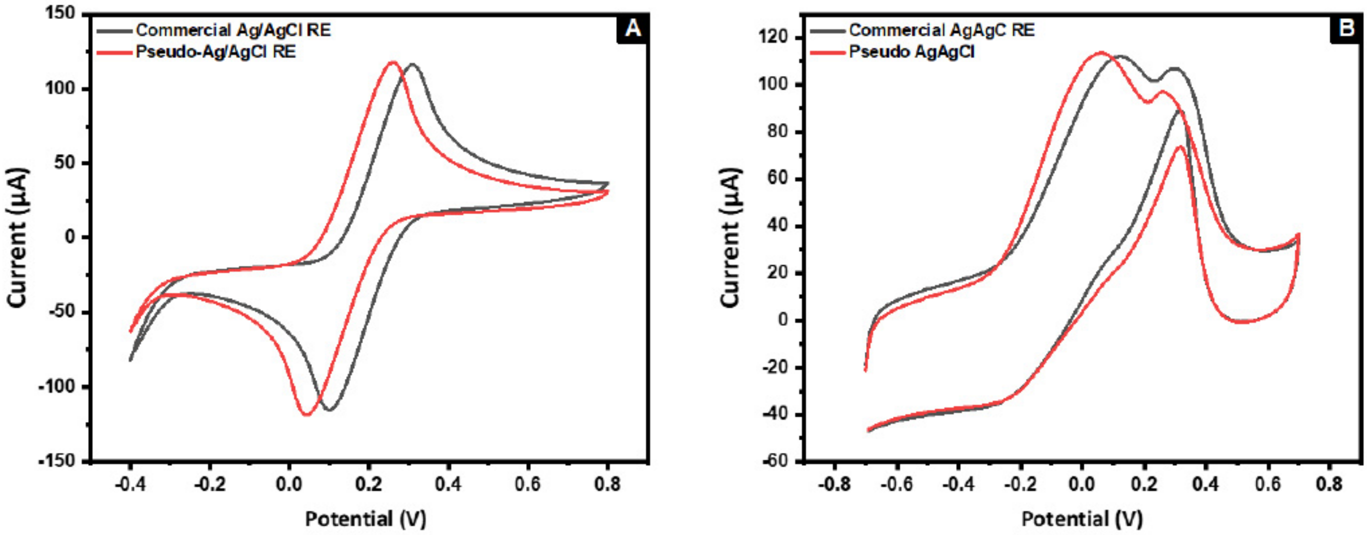

Figure S1. CV of 5 mM Ferricyanides redox at LSGE (**A**), CV of 10 mM glucose at AuNs-LSGE (**B**), with commercial Ag/AgCl RE (Black) and Pseudo-Ag/AgCl RE (Red), scan rate v = 50 mV·s^{-1}.

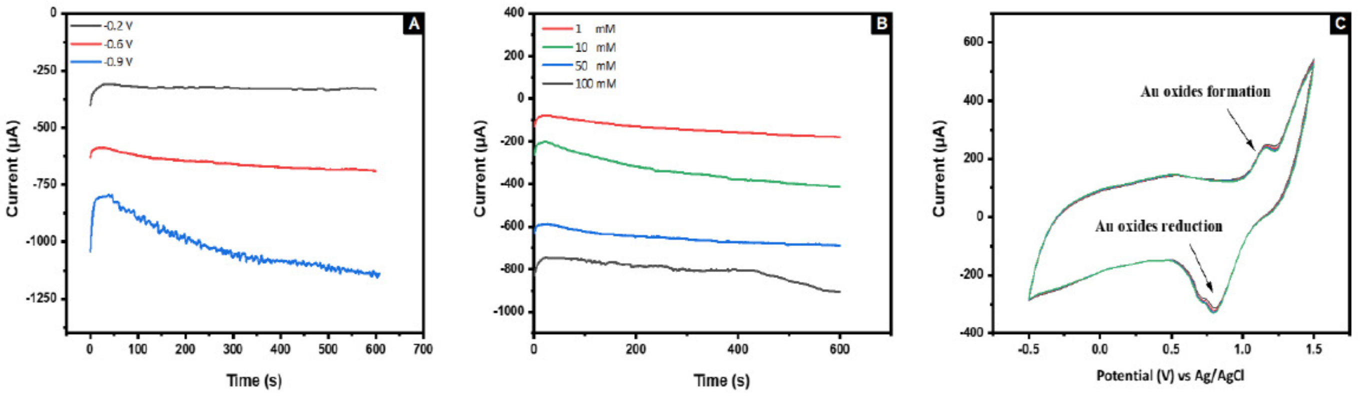

Figure S2. electrodeposition chronoamperograms of AuNs (**A**) at different potentials −0.2, −0.6, −0.9 V vs. Ag/AgCl (3M KCl) for 600 s in a 0.5M H_2SO_4, (**B**) at different gold precursor concentration 1, 10, 50 and 100 mM (**B**) five successive scans, related to the polarization of AuNs-LSGE performed by CVs in 0.5 M H_2SO_4 solution, scan rate 100 mV s^{-1}.

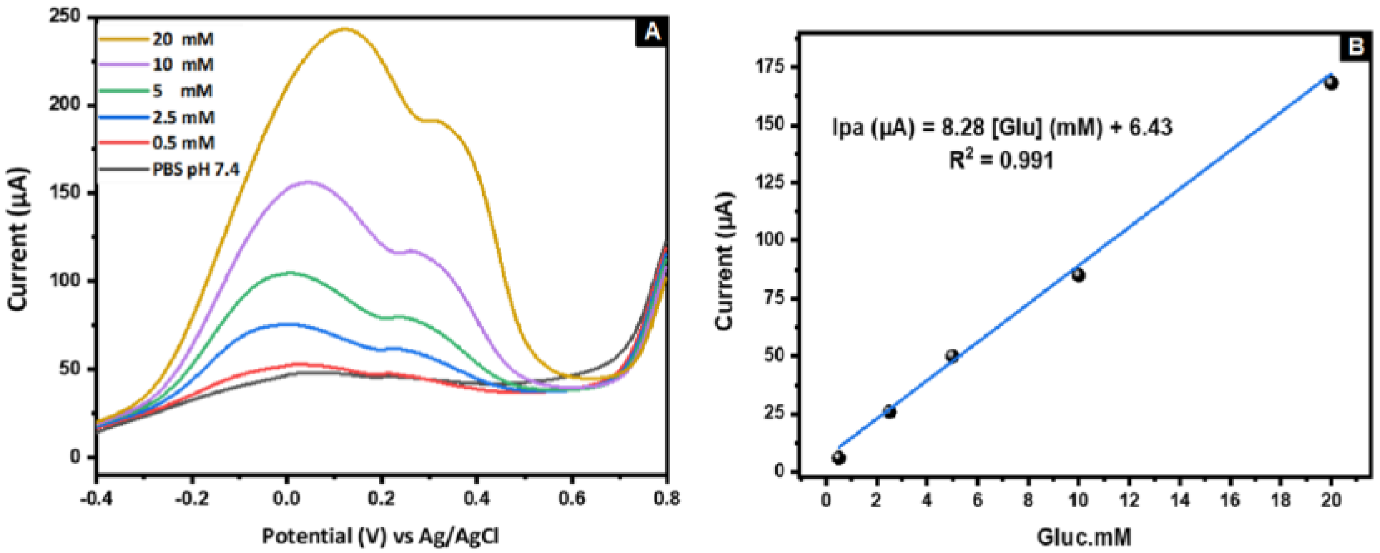

Figure S3. (**A**) LSV of glucose with different concentrations (0.5, 2.5, 5, 10, 20) at AuNs-LSGE in 0.1M PBS pH 7.4, scan rate; 50 mV·s^{-1} (**B**) The corresponding calibration curve.

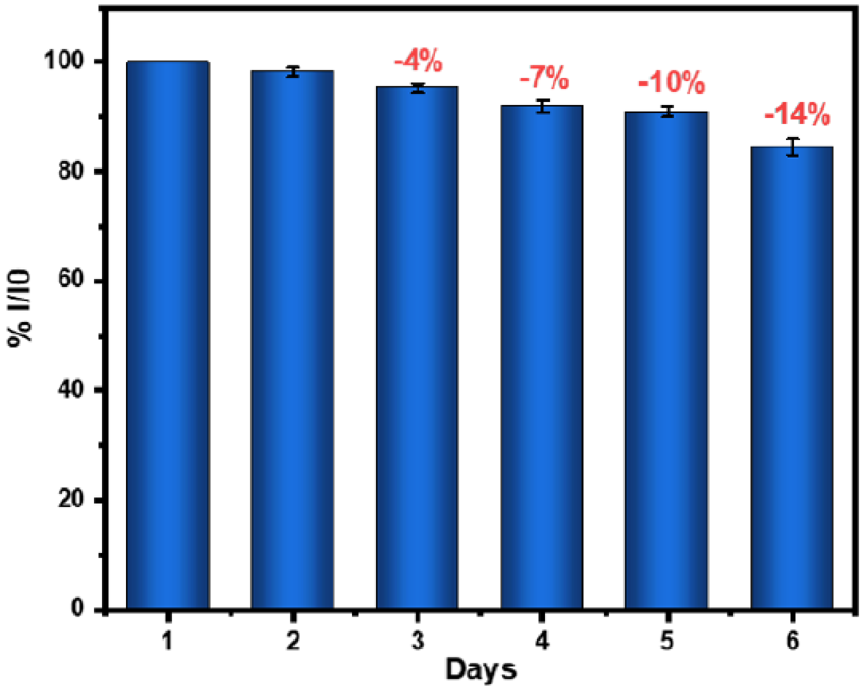

Figure S4. Current response % of 5 mM glucose over days recorded from I vs time (s).
